# PET Scans for Staging and Restaging in Diffuse Large B-Cell and Follicular Lymphomas

**DOI:** 10.1007/s11899-016-0318-1

**Published:** 2016-04-19

**Authors:** Sally F. Barrington, N. George Mikhaeel

**Affiliations:** PET Imaging Centre at St Thomas’ Hospital, Division of Imaging Sciences and Biomedical Engineering, King’s College London, Westminster Bridge Road, London, SE1 7EH UK; Department of Clinical Oncology, Guy’s and St Thomas’ NHS Foundation Trust, Guy’s Hospital, Great Maze Pond, London, SE1 9RT UK

**Keywords:** Positron emission tomography, Diffuse large B cell lymphoma, Follicular lymphoma, Diagnostic imaging, Computed tomography, Cancer staging

## Abstract

Positron emission tomography (PET)-CT was recommended in updated international guidelines for staging/restaging of diffuse large B-cell lymphoma (DLBCL) and follicular lymphoma (FL). In FL, PET was previously regarded as a research application only. This review concentrates on new publications related to PET in these diseases. In DLBCL, PET appears appropriate for staging using prognostic indices established with CT and baseline PET parameters, e.g. metabolic tumour volume, are prognostic of outcome. Early complete metabolic response (CMR) predicts end-of-treatment CMR with excellent prognosis. Patients without CMR at interim should not have treatment altered, but have a worse prognosis, and patients with other high risk features may need closer monitoring. The end-of-treatment scan is confirmed as the standard for remission assessment using Deauville criteria, which are also predictive for patients undergoing ASCT. In FL, PET is more sensitive for staging than CT but misses bone marrow involvement. PET-CT identifies patients at risk of progression after induction chemotherapy better than CT.

## Introduction

Positron emission tomography (PET) with 18F-fluorodeoxyglucose (FDG) has been used to image patients with diffuse large B-cell lymphoma (DLBCL) for over 25 years, whereas its potential utility in follicular lymphoma (FL) has only been appreciated more recently. Both are malignancies that are highly FDG-avid with uptake in more than 90–95 % of cases [1]. Changes in FDG uptake are frequently used for monitoring treatment both during chemotherapy and at completion of treatment. International guidelines [2••, 3••] support the use of PET-CT as the standard imaging modality for staging in DLBCL and FL and for remission assessment in DLBCL and in FL for patients undergoing immunochemotherapy.

However, the use of FDG-PET-CT continues to evolve with developments in the management of lymphoma, and this review will focus on these developments in DLBCL and FL. PET-CT provides more accurate staging than CT with better detection of all disease sites, but particularly extranodal sites, which are frequent in non-Hodgkin lymphoma (NHL). Accurate staging enables better prognostication, choice of therapy and comparison of results in clinical trials. We will review the additional value of PET-CT to already established prognostic indices. PET-CT is also more accurate than CT in remission assessment. In DLBCL where the aim of treatment is cure, an accurate assessment of remission is essential so that fit patients who are not in remission can be offered salvage therapy. This is not the same for FL, which is generally incurable. However, recent evidence suggests that complete remission assessed by PET-CT is strongly prognostic and may be used to guide intensity of follow-up or further treatment. Another feature of PET-CT is its ability to show metabolic response early during chemotherapy, which tends to correlate well with the final outcome of treatment. This can be used to select non-responding patients for a change in therapy and/or responding patients for potential de-escalation, as has been tested in HL. We review the current evidence for these approaches in DLBCL and FL.

## Diffuse Large B-Cell Lymphoma

DLBCL is the most common aggressive NHL worldwide and is curable in about 60–70 % of patients with a combination of anthracycline-containing chemotherapy and rituximab (e.g. RCHOP). For relapsed disease, high-dose chemotherapy and autologous stem-cell rescue is the standard treatment; however, it is not feasible in elderly patients with co-morbidities and in general seems to be less effective since the introduction of rituximab to first-line treatment. Pre-treatment prognosis is traditionally estimated using the International Prognostic Index (IPI) or one of its modifications, but response to first-line treatment is another very important prognostic factor.

### Staging—PET for Risk Stratification Pre-treatment

PET-CT is regarded as mandatory for staging patients with DLBCL because (i) CT alone understages patients by missing extranodal disease and (ii) a staging scan is required to identify initial disease sites when reporting end-of-treatment scans [2••]. Changes occur during treatment that need to be distinguished from lymphomatous involvement, which is evident on the staging scan. Changes may be FDG-avid but represent inflammation, infection and/or stimulation of normal marrow rather than lymphoma [4]. Recent publications have examined the effect of the more accurate staging with PET-CT on the prognostic ability of IPI [5, 6]. There is also growing interest in the significance of disease features on baseline PET, particularly in relation to the extent of extranodal involvement and total disease burden, both of which show promising prognostic value [7, 8, 9•].

### PET-CT Staging and Prognostic Indices

There are three prognostic indices available to risk-stratify patients: the IPI [10], the revised IPI (R-IPI) [11] introduced to account for improved prognosis after rituximab and the most recent National Comprehensive Cancer Network IPI (NCCN-IPI) [12] with further refinement based on grading some of the prognostic factors.

All use clinical factors - age, performance status (PS) and lactate dehydrogenase (LDH) level and imaging defined factors - Ann Arbor stage and extranodal involvement. These indexes were formulated using CT imaging. PET, now recommended for staging, upstages a significant proportion of patients, specifically detecting more extranodal disease, in particular with respect to bone marrow involvement [2••].

Recent studies examined the effect of PET upstaging on the performance of prognostic indices. El-Galaly and colleagues [5] evaluated stage, extranodal involvement and prognostic indices in 443 newly diagnosed patients with DLBCL staged with PET-CT, treated with R-CHOP or R-CHOP-‘like’ chemotherapy. PET-based IPI, R-IPI and NCCN-IPI were all predictive of outcome. Patients with very good (R-IPI) or low risk (NCCN-IPI) had excellent outcomes: 3y-PFS was 100 % (95 % confidence interval 88, 100) [*n* = 50] for very good risk and 3y-PFS and OS were 100 % (88, 100) [*n* = 54] for low-risk patients. However, the IPI and R-IPI failed to identify the group who are unlikely to be cured with RCHOP, with 3y-PFS >50 % for the poor risk group, but the NCCN-IPI was better at identifying this group (3y-PFS 33 % and OS 40 %).

### Significance of Extranodal Disease Extent

Another emerging theme in the study by El-Galaly et al. is the strong prognostic value of the extent of extranodal involvement. Two thirds of patients had extranodal disease on PET-CT, and significantly more treatment failures occurred with an increasing number of involved extranodal sites. By example, 3y-PFS was 25 % (7, 53) in patients with four or more extranodal sites compared to 79 % (71, 87) in patients with nodal disease only. Corresponding 3y-OS was 36 % (16, 56) vs 82 % (66, 98), respectively. Patients with extranodal involvement were older and had worse PS, more B symptoms and a higher prevalence of raised LDH. The extranodal sites associated with worse PFS and OS were bone marrow, pleura and gynaecological organs [13].

### Using Disease Burden/Metabolic Tumour Volume

**Fig. 1:**
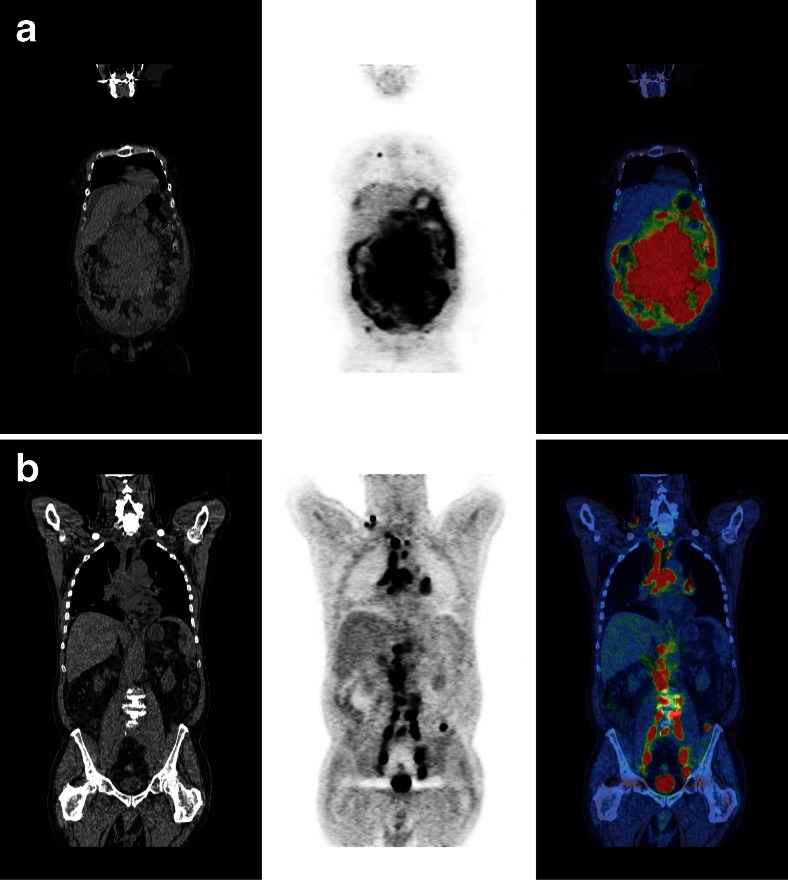
Coronal CT, PET and fused images are shown of patients with high metabolic tumour volume at baseline which is predictive of inferior prognosis. The patient in the *top panel* (**a**) had bulk disease by conventional assessment of maximal tumour dimension; the patient in the *bottom panel* (**b**) does not have bulky disease

Bulk is regarded as an adverse prognostic indicator in DLBCL, at least in patients with otherwise good prognosis [14]. It is commonly measured as the largest dimension of the largest mass. Metabolic tumour volume (MTV) is a more sophisticated, albeit time-consuming measurement of total disease burden based on volumetric measurement and metabolic activity. MTV is calculated by adding volumes of tumour, selected using a standardised uptake value (SUV) typically ≥2.5 or a percentage of the maximum SUV in areas of tumour [15, 16]. Recent publications report that MTV predicts prognosis [17, 18] better than bulk [9•]. An inherent problem, as with all measurements with a continuous distribution, is where to draw the cutoff between ‘high’ and ‘low’ MTV. A range of thresholds has been reported from 220 to 550 cm^3^, derived from receiver operating characteristic curves [9•, 17, 18]. The cutoff is influenced by the characteristics of the study population, with patients with earlier stage nodal disease only [17] having lower cutoffs than patients with high-risk more advanced disease [18]. Patients with low MTV tend to have better outcomes, with 3y-PFS 77–92 %, compared with patients with high MTV who have worse outcomes, 3y-PFS 48–56 % [9•, 17, 18], but again not sufficiently poor to consider treatment escalation. Mikhaeel et al. [9•] combined baseline MTV with early response assessment using Deauville scores at 2 cycles in 147 consecutive unselected patients treated for DLBCL with R-CHOP at a single institution. Patients could be separated into three distinct prognostic groups—good risk (low MTV regardless of PET-2 response), intermediate risk (high MTV, with CMR at 2 cycles) and poor risk (high MTV, no CMR at 2 cycles, Deauville scores 4, 5). 5y-PFS was >90 %, 58 % and 30 %, respectively (median FU = 3.8 years). The poor risk group contained 31 % of patients who experienced 58 % of the study events. These results demonstrate the previously unexplored interaction between pre-treatment prognosis and early response which enables better prognostication at an individual patient level. Validation of these results in larger prospective studies is warranted, as such an approach could improve on the prognostic power of interim PET (Fig. 1).

#### The Debate About Bone Marrow Biopsy

The debate about bone marrow biopsy in DLBCL continues to vex haemato-oncologists [19]. The high sensitivity of PET-CT for focal involvement in DLBCL in the bone marrow is well documented [20]. In two retrospective [21, 22] and one prospective [23] study involving 590 patients with newly diagnosed DLBCL, staged by PET-CT, no patients were changed to advanced stage based on bone marrow biopsy (BMB) alone, similar to findings in HL.

The contentious issue is whether the detection of low-volume involvement [24] (∼10–20 %) or the presence of discordant low-grade lymphoma in the marrow [25], which can be missed on PET, warrants BMB in all patients, or at least in patients with no evidence of bone marrow involvement on a staging PET-CT scan. Neither low-volume disease [26] nor indolent NHL [27] in the marrow has been demonstrated to affect outcome, independent of the IPI.

In the recent study by El-Galaly et al. [5] discussed above, 12/443 patients (3 %) had large cells in the marrow and 18 patients (4 %) had indolent NHL in the marrow when PET-CT scans did not demonstrate bone marrow involvement. This means that 26 patients with a ‘negative’ PET-CT scan for marrow involvement would need to undergo biopsy to detect a single case of missed large cells in the marrow. In a recent report by Cerci et al. [23], neither bone marrow involvement on PET-CT nor BMB alone adversely affected survival. Only patients with bone marrow involvement detected on both PET-CT and BMB at diagnosis had inferior prognosis, suggesting that disease burden in the marrow rather than marrow involvement per se influences prognosis. This can be readily appreciated by viewing the PET-CT scan in the multidisciplinary meeting. Nevertheless, haemato-oncologists who believe a BMB will influence patient management will wish to perform biopsy. We however advocate a selective approach, using BMB where results may influence prognosis or treatment, rather than routine biopsy in all patients [3••].

### Response Assessment—PET at Interim, End of Treatment and Prior to Autologous Stem Cell Transplant

Post-treatment remission assessment is important, as cure is the goal of treatment in DLBCL. PET-CT is the standard method in international guidelines [3••] and is widely used in routine practice. Nevertheless, several points are worth addressing:

1. What is the positive predictive value for end-of-treatment PET and is residual PET activity sufficient to initiate further or salvage treatment?
2. What is the prognosis of complete metabolic response (CMR) on end-of-treatment PET and is it independent of pre-treatment characteristics?
3. Can interim PET predict end-of-treatment PET result early?
4. Can interim PET be used to escalate treatment for poor responders?

Several studies have shown that residual activity in sites of previous disease on PET-CT, normally defined as Deauville score 4–5, is strongly predictive of residual disease. In R-CHOP-treated patients, progression-free survival (PFS) ranges from 24 to 35 % for positive end-of-treatment PET [28••, 29••]. This may be considered high enough to consider further treatment without biopsy confirmation. However, we would recommend that biopsy should be considered whenever possible to exclude the less common false-positive cases usually histologically reported as xanthomatous granulomatosis [2••]. Ultimately, the decision depends on the balance between the type of biopsy required (e.g. imaging-guided versus open surgery) and the intensity of treatment being considered (e.g. consolidation radiotherapy versus autologous stem cell transplant (ASCT)). Consideration of pre-treatment prognosis and response on interim imaging may also help the decision.

The negative predictive value of end-of-treatment PET is high, and most patients enjoy a long-term remission or cure. However, recent evidence also suggests that high-risk patients who achieve CMR still have a considerable risk of relapse. In a population-based study of 223 consecutive patients treated with R-CHOP-like immunochemotherapy [34], NCCN-IPI and R-IPI remained predictive of relapse irrespective of CT- or PET-defined remission status. Patients with NCCN-IPI 6–8 had a dismal outcome and higher risk of CNS relapse, whether or not they achieved CMR. The median age in this group, however, was 75 years. None responded to salvage treatment. In this elderly high-risk group, the authors suggested that alternative therapeutic approaches, e.g. novel agents, may be the only prospect for cure.

Early response assessment using interim PET is a more controversial area in DLBCL due to variable results reported in studies [30–37] and the debate on what action, if any, should be taken. We first examine the prognostic significance of interim PET negative and positive results and subsequently what action might be appropriate.

Studies demonstrate that 60–80 % of patients achieve CMR after 1–4 cycles of systemic therapy and tend to have an excellent PFS, usually in excess of 75–80 % [28••, 29••, 38]. More recent evidence also shows that achievement of early CMR predicts final CMR with very low risk of conversion to PET positivity (i.e. progression) after treatment [28••, 29••, 39].

Mamot el al. [28••] recently reported prospective results in 138 patients with DLBCL treated with R-CHOP-14 with response assessed using PET-CT. This important study employed standardised methods for PET with a common imaging protocol and strict observance of timing of scans in relation to chemotherapy. Treatment was not adapted according to interim PET, although patients with progression went off-study, suggesting that clinicians considered that it was unacceptable to continue with standard treatment in the presence of clear evidence of progression on interim PET, in line with recent international recommendations [2••]. There was a higher proportion of events than anticipated, likely because radiotherapy was included as an event, but the authors found similar results when using PFS as the end point. Analysis during the study used older International Harmonization Project reporting criteria [40], but a post hoc expert central review was performed using the five-point scale or Deauville criteria (DC) with scores 1–3 regarded as CMR. 2y-event-free survival (EFS) according to interim PET at 2 cycles was 75.9 % (63.7, 84.5) vs 41.4 % (28.7, 53.6) for patients with CMR and Deauville scores 4–5, respectively, *p* < 0.001. According to end-of-treatment PET, 2y-EFS was 71.5 % (61.2, 79.5) for CMR and 24.0 % (9.8, 41.7) for Deauville scores 4–5. Of note is that all patients with CMR on interim PET (60 %) remained in CMR at end of treatment and the EFS for a negative PET was very similar at interim and end of treatment.

Similar findings were reported in an earlier study [41] and a more recent study by Huntington et al. [39] where 79 % of patients had CMR at interim, all of whom had CMR at end of treatment. This confirms that interim PET shows CMR early in a significant proportion of patients and supports the premise that end-of-treatment scans are not necessary in patients who have CMR at interim.

Another interesting study was reported by Carr et al. [29••] combining interim (2–3 cycles) and end-of-treatment PET assessment from an international cohort of patients with DLBCL from disparate healthcare systems, sponsored by the International Atomic Energy Agency. They set a priori criteria which were similar, but not identical to DC. There was no significant difference in outcomes by country according to PET, suggesting that the technology is equally well applied in low- and high-income countries. In 327 patients, interim PET was negative in 64 % and positive in 36 %. The authors stratified 312 patients into four groups using the results of both interim PET and end-of-treatment PET. The best outcome was in the largest group (62 %) with interim and end-of-treatment CMR; 2y-EFS was 97 % (92, 98). Over half the patients who did not achieve CMR at interim had CMR at end of treatment (19 %), and these ‘slow’ responders, in whom bulky disease was more common, also had good outcomes, with 2y-EFS of 86 % (73, 93), although the hazard ratio (HR) for relapse compared to the previous group was 2.56 (1.08–6.11). These results imply that treatment escalation according to a positive interim PET could significantly over-treat many patients. Patients who had both positive interim PET and end-of-treatment PET (16 %) had 2y-EFS of 35 % (22, 48). Finally, 13 (4 %) patients progressed on treatment, with negative interim but positive end-of-treatment scans, 11 with biopsy-proven disease.

On the basis of these studies, it is clear that an early CMR on interim PET identifies a group with excellent prognosis, with the advantage of reassuring patients of the expected good outcome early. On the other hand, approximately half of patients with a positive interim PET will enter remission and change or escalation of treatment will over-treat an unacceptable proportion.

But more importantly is the fact that, to date, there has been no alternative treatment that proved to be superior to R-CHOP. In fact, studies which examined change or escalation on the basis of interim PET have not shown any benefit. Five studies have been reported either fully [42–44] or in abstract form [45, 46] using different PET criteria, timing and escalation strategies, summarised in Table 1. These studies demonstrated two findings: (1) the prognosis of interim PET positive is significantly worse, and (2) there was no improvement in this prognosis with changing therapy. The hope is that future research may produce better regimens which improve the outcome of patients not responding well to RCHOP. Until then, there is no justification for an early change in therapy unless there is definite evidence of progression.

**Table 1:** Summary of studies reporting response adaptation according to interim PET

Study	Patient population	Patient number	Interim PET timing	PET criteria	% PET positive	Intervention	Median FU	Overall outcome	End point
Pradal (GELTAMO) 2015	aaIPI > 1 or aaIPI = 1 + raised ß2M	71	>3 cycles MegaCHOP	IHP then ΔSUVmax 66 %	42 %	−ve: 3 MegaRCHOP +ve: 2 RICE + BEAM ASCT (no info on RT)	42.8 m	4y-PFS 67 % OS 78 %	3y-PFS −ve: 81 %, +ve: 57 % OS −ve: 95 %, +ve 33 %
Swinnen (ECOG) 2014	Stage 3–4 or 2 with >10 cm mass	74	>3 RCHOP	IHP/London	16 %	−ve: 3 RCHOP +ve: 4 RICE (no RT)	54 m	4y-PFS 64 % OS 86 %	2y-PFS −ve: 76 %, +ve: 42 % OS −ve: 93 %, +ve 69 %
Duehrsen (PETAL) 2014	80 % DLBCL	853 (926)	>2 RCHOP	ΔSUVmax 66 %	13 %	−ve: randomise to 4 RCHOP or 4 RCHOP + 2R +ve: randomise to 4 RCHOP or intensification	33 m	2y-TTF −ve: 79 % +ve: 47 %	2 R: no difference (HR 1.2, 95 % CI 0.8–2.1) Intensification: no difference (HR 1.6, 95 % CI 0.9–2.7)
Sehn (BCCA) 2014	Stage 3–4 or 2 with B symptoms or >10 cm mass	155	>4 RCHOP	IHP	33 %	−ve: 2 RCHOP +ve: 4 RICE + RT for EOT PET + ve		4y-PFS 79 % OS 87 %	4y-PFS −ve: 91 %, +ve: 59 % OS −ve: 96 %, +ve 73 %
Kasamon 2009	98 % B-cell NHL	59	>2–3 RCHOP	5-point scale	56 %	−ve: continue RCHOP +ve: 2 ESHAP or ICE + ASCT RT permissible	33.6 m	2y-EFS 77 % OS 82 %	2y-EFS −ve: 89 %, +ve: 67 %

*aaIPI* age adjusted International Prognostic Index, *ASCT* autologous stem cell transplantation, *BCCA* British Columbia Cancer Agency, *BEAM* carmustine or lomustine etoposide, cytarabine, melphalan, *ß2M* beta 2 micoglobulin, *ΔSUVmax* change in maximum standardised uptake value, *ECOG* Eastern Cooperative Group, *EOT* end-of-treatment, *EFS* event-free survival, *GELTAMO* Grupo Español de Linfomas y Trasplantes de Médula Ósea (Spanish group of Lymphoma and Bone Marrow Transplantation), *HR* hazard ratio, *IHP* international harmonization project, *OS* overall survival, *PFS* progression-free survival, *RCHOP* cyclophosphamide, doxorubicin, vincristine prednisolone, *RICE* rituximab, ifosfamide, carboplatin, etoposide, *TTF* time to treatment failure

So where does this leave the role of interim PET in DLBCL? We assert that it is an excellent negative test, showing remission early and enabling reassurance of patients whilst still having treatment. In addition, it may show lack of any response or early progression in a small proportion of patients. Is it essential? The answer is no, but if interim imaging is performed, we would recommend PET-CT in preference to CT for the above reasons and recommend that if the result shows CMR then an end-of-treatment-PET is not required, which saves resources and inconvenience. On the other hand, if the result is positive, there is no indication to change treatment, but close monitoring of these patients during treatment may be warranted, as a proportion of them may progress. In our practice, if the initial disease was poor risk (e.g. high IPI) and PET-2 is positive, we prefer to monitor the patient with repeat PET after 4 cycles of treatment [9•].

#### PET for Pre-transplant Assessment

Various studies have reported that PET is predictive of outcomes following high-dose chemotherapy before ASCT [47–49], prior to DC, which are recommended by current guidelines. Sauter et al. [50•] reported a retrospective analysis of 129 patients with B-NHL, two thirds with DLBCL, who underwent ASCT based on at least partial response using CT. Scans were scored using DC. Patients with CMR and Deauville scores 1–3 had 3y-PFS of 77 % and 3y-OS of 86 % compared to patients with Deauville scores ≥4 with 3y-PFS of 49 % and 3y-OS for 54 %, leading the authors to conclude that patients with inadequate response on PET-CT should be the focus of risk-adapted investigational therapies.

## Follicular Lymphoma

FL is the second most common lymphoma type worldwide, and although generally characterised by an indolent course, it has a very varied natural history. With significant changes in its management, particularly the introduction of monoclonal antibodies and improvements in prognosis and overall survival, the paradigm of treatment is shifting from symptom palliation to more active treatment with the aim of prolonging remission and survival. Imaging modalities and accurate remission assessment are therefore becoming more important.

In early stage disease, radiotherapy (RT) remains the standard treatment resulting in durable remissions and possibly cure in almost half of patients staged without PET. Most relapses occur outside the radiation field, indicating failure of initial staging rather than RT. Therefore, more accurate staging could help improve selection for RT and reduce the incidence of relapse [51, 52•, 53, 54].

For advanced disease, watch and wait remains an option for low-volume asymptomatic disease. Accurate assessment of disease extent is important in this case, and criteria have been developed to select patients suitable for this approach [55]. For patients with higher disease burden or symptoms, the current standard approach is chemo-immunotherapy induction followed by 2 years of maintenance rituximab which results in a long PFS for most patients. However, there are probably a substantial number of patients who do not benefit from prolonged maintenance and there is a small group of patients (about 20 %) who relapse early within 2 years, even after anthracycline-containing induction, and have a much shorter OS [56]. Identifying those patients is important as they need alternative treatment approaches. New therapeutic options have become available in the last decade including new antibodies and agents targeting oncogenic pathways [57].

### PET for Pre-treatment Staging

PET-CT detects more nodal and extranodal sites than CT in patients with FL undergoing induction chemotherapy [52•]. The impact on staging is higher in patients with apparently limited stage on CT in whom PET is performed (i) to determine if local RT treatment is appropriate (ii) to plan RT fields. In a recent phase III prospective study which randomised patients to one of three R-chemotherapy treatments, PET-CT altered the Follicular Lymphoma International Prognostic Index (FLIPI) score in 35/142 patients, increasing the score in 18 % of patients overall and in 62 % of patients (15/24) with limited disease on CT [52•], confirming earlier reports [51, 53, 54]. Although outcomes from patients selected for RT using PET-CT have not been published, this would appear to be a sensible indication for PET-CT in patients with FL. Bone marrow biopsy detected bone marrow involvement in 46/108 (43 %) of patients without marrow abnormalities on PET [52•], indicating that BMB is required for diagnostic workup. The ability of PET to select sites for biopsy in cases with suspected transformation has also been previously described [58, 59] and is recommended in current guidelines [2••].

### Response Assessment—PET at Interim, End of Treatment and Prior to ASCT

PET-CT was initially supported for response assessment in the research setting [60] but has now become the standard imaging modality [2••, 3••]. This was based on three multicentre studies where PET-CT was used to assess response in patients with high tumour burden symptomatic FL or advanced disease, which included 122 [61], 112 [62] and 205 [63] patients, respectively. All reported end-of-treatment PET-CT to be predictive of PFS, independent of the FLIPI and superior to CT-based response. Interim PET (cycle 4) was predictive of PFS, but not as strongly predictive as the end-of-treatment scan in the prospective PET-Folliculaire study [62].

A pooled analysis of PET-CT response in the three studies, using central scan review and DC, was published in 2014 [64••]. The analysis included 246 patients with PET-CT scans available for review. Median FU was 54.8 m. Seventy-three percent of patients were treated with R-CHOP, 15 % with R-CVP and 12 % with R-FM. Eighty-three percent of patients had a negative scan (Deauville scores 1–3). The study revealed three important findings. Firstly, there was a significant number of patients who had their response re-classified with PET compared to CT-based International Working Group (IWC) criteria. Of 128 patients with CR/CRu, 17 (13 %) had Deauville scores 4–5, and of 72 with PR/SD/PD, 50 (69 %) were re-classified as CMR (Deauville scores 1–3). Secondly, the PET-based response was more predictive of PFS and OS than IWC, in the whole group, in the RCHOP-treated patients and in the responding patients according to IWC. In the whole group, 4y-PFS was 63.4 % (55.9, 70.0) for patients with CMR, compared with 23.2 % (11.1, 37.9) for patients without CMR [HR 3.9 (2.5,5.9) *p* < 0.0001]. The difference in median PFS was very large: 74 and 16.9 months for patients with scans showing CMR and no CMR, respectively. Finally, PET-based response was an independent and stronger predictor of PFS than FLIPI and IWC response.

These data establish PET-CT as the imaging modality of choice for remission assessment in FL, but more importantly, it shows that PET response using DC can identify the small group of patients who are likely to have an early progression (median PFS 16.9 m). As discussed in the “Introduction” section, it has been shown that patients who progress within 2 years have worse OS (5y-OS 50 % compared to 90 % for longer remission) [56] who may be candidates for close monitoring and testing different treatment approaches if they relapse early. Patients with CMR can be confidently reassured about the prospect of long PFS. The discriminatory ability of PET response to first-line therapy in FL lends itself to being used in studies testing response-adapted therapy, further refining the management of this disease with varied natural history.

Lastly, salvage treatment and ASCT is an option for patients with refractory or relapsed disease who are sufficiently fit. An initial report from the Lymphoma Studies Association in 59 patients with relapsed/refractory disease after first-line R-CHOP suggested that PET may be able to predict response following high-dose chemotherapy prior to ASCT [65], as previously reported in HL and DLBCL.

## Conclusions (Table 2)

In DLBCL:

1. PET-CT can be used with prognostic indices to risk-stratify patients [5]. Poor risk is best predicted using the NCCN-IPI [5, 6], but this group consists of elderly patients unfit for salvage treatments in whom novel agents may be explored [6].
2. The number of extranodal sites, including bone marrow involvement [5] and disease burden using MTV [9•, 17, 18], are promising baseline predictor of prognosis using PET, which can be combined with early response assessment [9•].
3. CMR on interim PET is predictive of excellent prognosis and allows patients on treatment to be reassured. Such patients do not require end-of-treatment PET [28••, 29••, 38, 39]. Treatment escalation, however, when patients do not have early CMR, is unjustified [29••, 42–46], but closer monitoring, especially for patients with other high risk features, may be appropriate.
4. End-of-treatment PET is better for remission assessment than CT. Patients who do not achieve end-of-treatment CMR should be considered for further treatment after biopsy confirmation, where feasible [28••].

In FL:

5. PET-CT is more sensitive for staging than CT [52•] and can be used to select biopsy sites in clinically suspected transformation [58, 59].
6. PET-CT identifies patients at increased risk of early progression following induction chemotherapy with R-CHOP(like) therapy [64••] and would be suitable to select patients for response-adapted trials testing new agents.

**Table 2:** Role for PET-CT in staging and restaging patients with DLBCL and FL

DLBCL	FL
Baseline PET used for:	
Risk stratification	Risk stratification
*NCCN-IPI using PET discriminates patients with very good prognosis from patients at high risk of treatment failure, mostly elderly patients unsuitable for salvage treatments for whom testing with novel agents may be appropriate*	*PET-CT upstages patients compared to CT; effect on outcomes not known*
*Parameters including number of extranodal sites and metabolic tumour burden, also combined with early response are promising predictors of prognosis.*	
Staging including bone marrow assessment	Staging
*Can replace bone marrow biopsy in selected cases*	*To assess suitability for local (RT) or systemic treatment low sensitivity for bone marrow assessment; bone marrow biopsy required*
Mapping initial disease sites for accurate response assessment	Mapping initial disease sites for accurate response assessment
*Differentiating lymphomatous involvement from other causes for increased FDG uptake, e.g. infection, inflammation, bone marrow hyperplasia*	*Differentiating lymphomatous involvement from other causes for increased FDG uptake, e.g. infection, inflammation, bone marrow hyperplasia*
Interim PET used for:	
Prognosis	*Prognostic but no current role*
*Early CMR has excellent prognosis and usually predicts CMR at end of treatment; such patients do not require end-of-treatment scans.*	
*Patients with a positive interim PET and other high risk features, e.g. poor-risk IPI, may require close monitoring during treatment as they have higher risk of refractory disease and relapse.*	
*PET is a more appropriate test for interim imaging assessment than CT.*	
Excluding disease progression on treatment	
*But should not be used to change standard treatment unless clear evidence of progression. To date, no evidence exists that response adaptation at interim on the basis of positive PET improves patient outcomes and risks over-treating many patients.*	
End of treatment PET used for:	
Remission assessment	Remission assessment
*Using Deauville criteria. Patients with end-of-treatment Deauville scores 4 and 5 should be considered for further treatment with biopsy confirmation wherever feasible but particularly if salvage treatment ± ASCT is being considered.*	*After induction treatment with R-CHOP(-like) chemotherapy using Deauville criteria. Patients with end-of-treatment Deauville scores 4 and 5 have worse outcomes than patients achieving CMR and may be suitable for testing of response-adapted strategies.*
Decision making as to suitability for ASCT following high-dose chemotherapy	*Early data suggest may be predictive of outcomes after following high-dose chemotherapy prior to ASCT.*
*In preference to CT*	
